# Validation of the digital health literacy assessment among the university students in China

**DOI:** 10.3389/fpubh.2023.1294183

**Published:** 2024-01-18

**Authors:** Limei Nie, Jiajia Zhao, Lutong Pan, Mingli Pang, Jieru Wang, Yue Zhou, Rui Chen, Hui Liu, Xixing Xu, Baochen Su, Fanlei Kong

**Affiliations:** ^1^Centre for Health Management and Policy Research, School of Public Health, Cheeloo College of Medicine, Shandong University, Jinan, China; ^2^NHC Key Lab of Health Economics and Policy Research, Shandong University, Jinan, China; ^3^Institute of Health and Elderly Care, Shandong University, Jinan, China; ^4^Department of Mathematics, College of Art and Science, New York University, New York City, NY, United States

**Keywords:** digital health literacy assessment, reliability, validity, university students, China

## Abstract

**Purpose:**

With the development of the internet, digital health literacy (DHL) has become increasingly important for managing health. Consequently, various digital health literacy scales have been created for different groups. The purpose of this study was to verify the reliability and validity of the simplified Chinese version of the Digital Health Literacy Assessment (DHLA) scale among university students in China.

**Method:**

Snowball sampling was used to recruit the participants via an online platform (Wenjuan.com), and finally 304 university students were included in the survey. Demographic information and the status of DHL were collected through the online questionnaire. Cronbach’s alpha and split-half reliability were used to test the internal consistency of the scale, while the structural validity was verified by exploratory factor analysis and confirmatory factor analysis. Additionally, the convergence of the scale was tested by composite reliability (CR) and average variance extracted (AVE).

**Result:**

Two dimensions were generated from 10 entries in the scale, named Self-rated Digital Health Literacy and Trust Degree of Online Health Information, respectively. The Cronbach’s alpha and split-half reliability of the total scale were 0.912 and 0.828, while the Cronbach’s alpha of the two dimensions were 0.913 and 0.830, respectively. The structural validity-related indexes of the scale met the standards (RMSEA = 0.079, GFI = 0.943, AGFI = 0.902, CFI = 0.971). In each dimension, the CR and AVE also reached critical values (CR > 0.7 and AVE > 0.5).

**Conclusion:**

The scale had high reliability and validity, indicating the simplified Chinese DHLA scale could be used to evaluate the DHL of university students in China.

## 1 Introduction

The rapid development of information technology has made the internet an accessible resource for people to obtain health information (1). The size of China’s internet users is 1.067 billion and the internet penetration rate has reached 75.6% in 2023 (2), of which, the internet usage rate of university students has reached nearly 100% (3). University students use online health information to address or solve health problems and communicate about their health issues online (4), yet health misinformation is rife on social media (5). As a vulnerable group, university students may lack knowledge and skills for seeking and evaluating health information from the internet (6), which makes the research on the improvement of digital health literacy (DHL) among university students more and more important.

In 2006, Norman and Skinner first defined electronic health literacy (eHL) as the ability to read, use computers, search for information, understand health information, and put it into context (7). With the innovative development of digital technology, interactivity on the Web has become more and more important, the concept of DHL was then introduced in 2021 (8), where DHL refers to the skills to search, select, evaluate, and apply online health information and healthcare-related digital applications (9, 10). Compared to eHL’s focus solely on the ability to read and send information online, DHL further includes the skills of writing and communicating health-related messages online; that is how DHL differs from eHL and emphasizes people’s interactivity with the internet (10, 11).

With the digitization of health care and the wide availability of Web-based applications, DHL is an essential skill to be mastered in the digital age, which is an important determinant of health (12) and has a profound impact on an individual’s health (13). Existing research has shown that DHL could alleviate anxiety related to both physical health and the usage of digital technology among older adults (14). Additionally, DHL was also found to be related to the health status of patients with cancer (15) and cardiovascular disease (16). Moreover, DHL could enhance patient autonomy and improve the doctor–patient relationship by ensuring that patients use online health information correctly (17, 18). Digital technologies could increase the transparency of health information, yet could also hinder access to health information due to low DHL (19). Therefore, it is crucial to use scales to assess the DHL of the population promptly and implement intervention measures for people with low literacy levels.

However, few studies have focused on the assessment tools of DHL currently (20). The tools for assessing DHL include the Digital Health Literacy Assessment (DHLA) (21), Digital Health Literacy Instrument (DHLI) (10), Digital Health Technology Literacy Assessment Questionnaire (DHTL-AQ) (22), and Digital Health Literacy Assessment Scale for Community-dwelling older adults (23). The traditional Chinese version of the DHLA was developed by Peggy Liu based on the eHealth Literacy Scale (eHEALS), which has 10 items (three dimensions) and good reliability and validity in Taiwan Province, China. Moreover, a strong correlation between the total score of the DHLA and DHLI also indicated that the DHLA could be used to measure DHL (21). To date, no research has ever tested the validation of the DHLA among the population in mainland China; considering the vulnerability of university students and their frequent use of the internet, this study chose them as the target population. Thus, the purpose of this study was to verify the reliability and validity of the simplified Chinese DHLA among university students in mainland China.

## 2 Materials and methods

### 2.1 Study design and participants

This study utilized a cross-sectional correlational design using a self-assessment questionnaire. Snowball sampling was used to recruit the participants. An online questionnaire was created from an online platform (Wenjuan.com) and distributed to university students in China through WeChat to collect the data. The survey began on 8 September 2022 and ended on 17 September 2022. Ultimately, a total of 304 participants from nine Chinese provinces were selected and interviewed. However, 22 participants were excluded as they answered their questionnaires incorrectly or incompletely, including those with incomplete answers (five questionnaires), took less than 3 min to complete the questionnaire (11 questionnaires), or their age was under 10 years (six questionnaires). Ultimately, a total of 282 participants were finally included in the data analysis.

### 2.2 Measurement

Based on the traditional Chinese version of the DHLA, the simplified Chinese version of the DHLA was created and used to assess the DHL of university students in China. The original scale had 10 questions and was divided into three dimensions, with each question being answered using a 5-point Likert scale. The first to sixth questions belonged to the first dimension and were entitled Self-rated digital health literacy, with response styles ranging from 1 (very bad) to 5 (very good). The seventh to ninth questions belonged to the second dimension and were named as the Trust degree of online health information. The tenth question belonged to the third dimension and was named as the Trust degree of online traditional Chinese medicine health information. The options for the seventh to tenth question ranged from 1 (very unconvinced) to 5 (very convinced).

Besides the DHLA, the questionnaire also included: (1) sociodemographic characteristics of the participants: sex, age, educational level, and residential area; and (2) eHEALS.

### 2.3 Statistical analysis

Descriptive analyses, Chi-square test, and ANOVA were conducted to explore the characteristics and distribution differences between male and female university students. A *p-*value <0.01 denotes statistical significance. The critical indicators of the quality of a measuring instrument are reliability and validity (24). Depending on the type of questionnaire, some validity tests are mandatory to apply (such as the internal consistency reliability, construct validity, or construct convergent validity) (25). In this study, internal consistency reliability, construct validity, convergent validity, and criterion validity were chosen to verify the quality of the DHLA. All analyses were performed using the Statistical Package for Social Sciences (SPSS, IBM Corp. Released 2016. IBM SPSS Statistics for Windows, Version 24.0. IBM Corp., Armonk, NY, United States).

#### 2.3.1 Distributional properties of the scale

The study used the Kolmogorov–Smirnov (KS) test which is a well-known non-parametric goodness-of-fit test to check whether the scale scores conformed to the normal distribution (26). Floor or ceiling effects are considered to be present if more than 15% of respondents achieved the lowest or highest score, respectively (27). The items of the scale were judged comprehensively according to the screening criteria of item analysis: item–scale correlation ≥0.4 and Cronbach’s α does not increase if the item deleted. (28).

#### 2.3.2 Reliability

The reliability of the DHLA was tested using Cronbach’s alpha and split-half reliability. The Cronbach’s alpha α >0.8 (29) and split-half reliability >0.85 (30) indicated good internal consistency of the scale in this study. The Spearman–Brown formula was then used to analyze the split-half reliability to compare with Cronbach’s alpha. When the Cronbach’s alpha α >0.8 and the value of split-half reliability >0.85, the scale was prove to have good reliability. (31).

#### 2.3.3 Construct validity

The Kaiser-Meyer-Olkin (KMO) measure and Bartlett’s test were used to test the suitability of the data for the exploratory factor analysis (EFA). This study used a KMO of ≥0.8 and a significant Bartlett’s test *p* < 0.05 as empirical evidence of a sufficiently large sample size for factor analysis (32, 33).

Since the DHLA is a multiple factor scale, a confirmatory factor analysis (CFA) was then conducted to investigate its construct validity by factor structure model (34). CFA is an old and mature method of confirming the number of factors in a scale to test how well the data fits the proposed model (35). A χ^2^/df < 3.00, root mean square error of approximation (RMSEA) < 0.08, goodness-of-fit index (GFI) > 0.900, adjusted goodness of fit index (AGFI) > 0.900, and comparative fit index (CFI) > 0.900 indicated a reasonable fit (36).

#### 2.3.4 Convergent validity

Convergent validity is the illustration of substantial and significant correlation between different scales designed to assess a common construct, which is a subset of construct validity and regarded as a core component of the validity in a test (37). To test the correlation between factors of the DHLA, the average variance extracted (AVE) and composite reliability (CR) were used to evaluate the convergent validity of the scale. The AVE > 0.5 and CR > 0.7 indicated the convergent validity of the scale is acceptable (38).

#### 2.3.5 Criterion validity

Criterion validity can reflect the degree of agreement between a measured score and an external criterion. Finding the relevant, valid, objective, uncontaminated, and practical criterion and measuring the criterion accurately is the basis of the test (39). This study chose eHEALS which is the most commonly used method to assess DHL and assess whether individuals can utilize e-healthcare resources actively (40), and the correlation method was used to estimate the criterion validity. The larger the correlation coefficient r, the higher the correlation degree between the score and criterion, and the scale can better measure or predict the content of the study.

### 2.4 Ethical considerations

All the participants provided informed consent for inclusion in the study. This study was approved by the Shandong University Institutional Ethics Committee (task no. LL20220425).

## 3 Results

### 3.1 Characteristics of participants (*N* = 304)

Table 1 shows the demographic characteristics of the participants. The majority of them (97.5%) were 19 years old or older, with 203 (72.0%) being female and 79 (28.0%) male. In addition, 67% (189) of the students were living in cities, and almost half (53.2%) of the participants were pursuing undergraduate programs. There were no statistically significant differences (P>0.05) between the male and female university students in age, residential area, and educational level.

**Table 1:** Descriptive statistics of participants.

Characteristics	Categories	Total	Male	Female	*P*
		N (%)	N (%)	N (%)	
Observations		282 (100)	79 (28.0)	203 (72.0)	
Age	<=18	7 (2.5)	3 (42.9)	4 (57.1)	0.648
	19 ~ 22	130 (46.1)	37 (28.5)	93 (71.5)	
	> = 23	145 (51.4)	39 (26.9)	106 (73.1)	
Residential area	City	189 (67.0)	57 (72.2)	132 (65.0)	0.264
	Rural	93 (33.0)	22 (27.8)	71 (35.0)	
Educational level	First and Second Undergraduate	30 (10.6)	8 (10.1)	22 (10.8)	0.670
	Third, Fourth, and Fifth Undergraduate	120 (42.6)	37 (46.8)	83 (40.9)	
	Masters and Higher	132 (46.8)	34 (43.0)	98 (48.3)	

### 3.2 Distributional properties of the DHLA

The total score on the scale ranges from 10 to 50 points. Through exploratory analysis, the Kolmogorov–Smirnov test was significant (*p* < 0.05), indicating that the scores were not normally distributed. In this study, 13 (4.6%) subjects scored the highest score and 0 subjects scored the lowest (both under 15%) indicating that the DHLA had no significant floor or ceiling effect. Table 2 shows the item analysis of the scale. Cronbach’s α remained hardly increased when any one of the items was deleted from the calculation except for item 10. Meanwhile, the item–total correlation coefficients were high, with individual item values ranging from 0.585 to 0.843 (*p* < 0.01 for each one of the correlations).

**Table 2:** Reliability analysis of internal consistency in the DHLA.

Item	Mean (SD)	Item–scale correlation	Cronbach’s α if item deleted
Scale	37.55 (0.414)		
SRDHL1	4.23 (0.720)	0.633*	0.909
SRDHL2	4.05 (0.790)	0.809*	0.899
SRDHL3	3.99 (0.829)	0.843*	0.896
SRDHL4	3.91 (0.860)	0.799*	0.899
SRDHL5	3.73 (0.968)	0.799*	0.899
SRDHL6	3.77 (0.892)	0.784*	0.900
TDOHI1	3.65 (0.913)	0.760*	0.902
TDOHI2	3.68 (0.896)	0.808*	0.899
TDOHI3	3.72 (0.861)	0.744*	0.903
TDOHI4	2.82 (1.261)	0.585*	0.923

* means *p* < 0.01, SRDHL, Self-rated Digital Health Literacy; TDOHI, Trust Degree of Online Health Information.

### 3.3 Reliability

The results of the reliability tests for the total and two-dimensional scores of the DHLA are displayed in Table 2. In this study, the α of all the 10 items was 0.912, and the split-half reliability was 0.828, both of which were higher than the standard value of 0.8. Additionally, the α for each dimension was above 0.8.

### 3.4 Validity

#### 3.4.1 Construct validity

**Figure 1:**
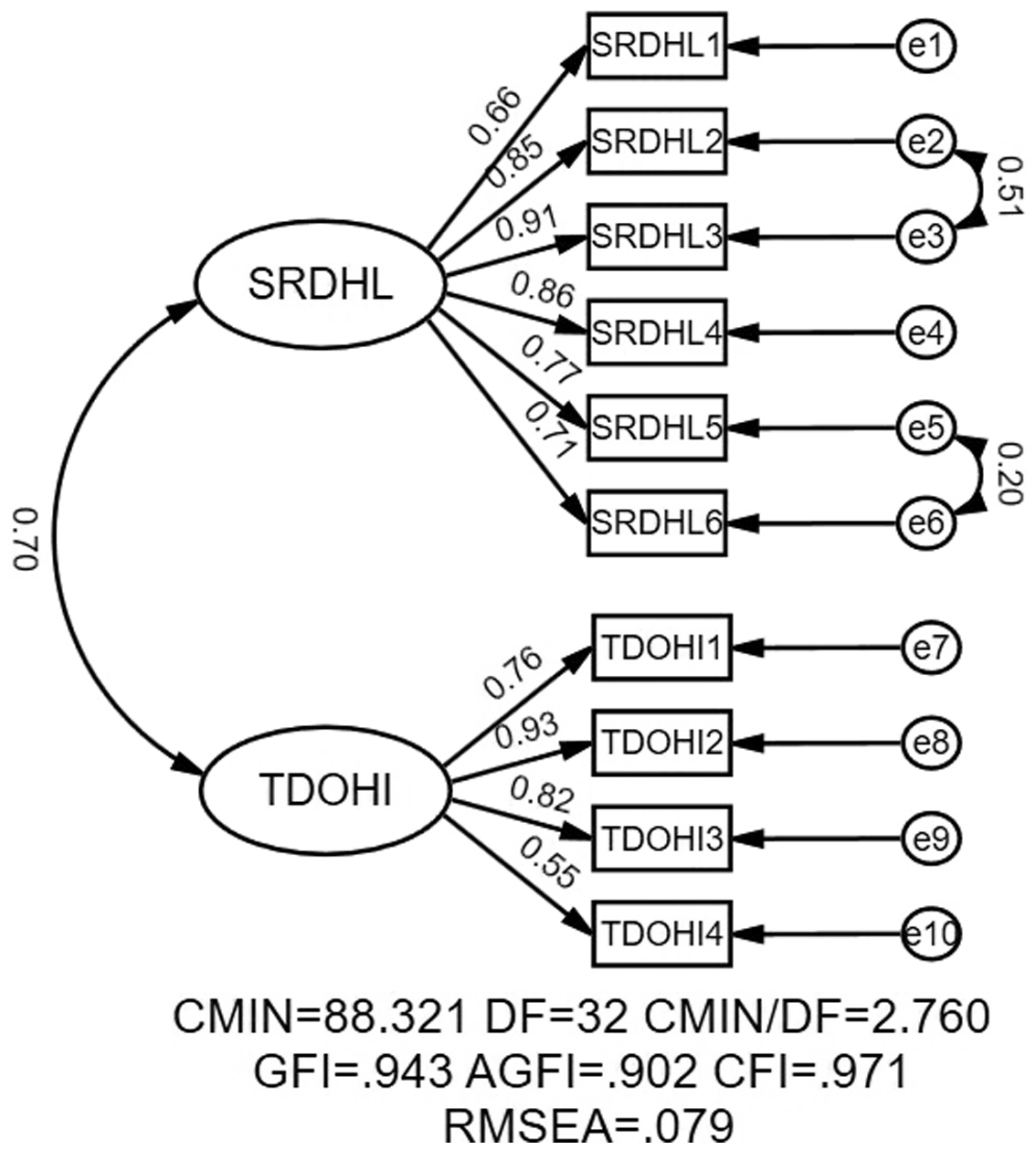
The measurement model. SRDHL, Self-rated Digital Health Literacy; TDOHI, Trust Degree of Online Health Information.

In this study, KMO = 0.906, and Bartlett’s test significance level was *p* < 0.01. The exploratory factor analysis of the 10 items of the DHLA showed that it was suitable for factor analysis. The principal component analysis method was used to extract common factors with an eigenvalue >1 by the maximum variance method. Considered together with the result of the steep slope map test, it was more appropriate to retain the two factors, which was different from the results of the original scale with three factors in Taiwan on the factor classification. After analyzing various dimensionality reduction methods, most of the data results showed that factor 2 (network information trust) and factor 3 (folk information trust) of the original scale should be combined as one factor (named “network health information trust”) according to the database used in this study. Ultimately, there were two dimensions in the whole scale, with the first to sixth questions entitled “Self-rated Digital Health Literacy” (shorted for SRDHL), and the seventh to tenth questions entitled “Trust Degree of Online Health Information” (shorted for TDOHI). The dimensionality factor loading ranged from 0.621 to 0.884. To further verify the scale structure validity, AMOS software was used for two-factor structure validation analysis. According to the correction index, a total of two covariant relations between errors were added and the modified model fitting indexes all reached the reference standard, which indicated that the model was well-fitted. Multiple criteria in the CFA analysis showed a good fit to the two-factor structural model: RMSEA = 0.079, GFI = 0.943, AGFI = 0.902, and CFI = 0.971 (as shown in Figure 1).

#### 3.4.2 Convergent validity

Table 3 shows that the CR and AVE values were conducted to examine the convergent validity of SRDHL and TDOHI. Both dimensions met the standard AVE > 0.50 and CR > 0.70. The Chinese version of the DHLA further proved the convergence efficiency. The values of standardized factor load were also obtained. Factor loading of each item in the scale was greater than 0.5 (Table 4).

**Table 3:** Descriptive statistics and reliability analysis of three dimensions and total scores.

	Mean	SD	Skewness	Kurtosis	α	Split-Half
Total 10 items	37.55	0.414	−0.218	0.549	0.912	0.828
Self-rated Digital Health Literacy	23.68	0.256	−0.397	0.333	0.913	0.876
Trust Degree of Online Health Information	13.87	0.199	0.021	−0.221	0.830	0.848

SD, Standard Deviation.

**Table 4:** CR and AVE values for the two dimensions.

Dimension	Items	Standardized factor loading	CR	AVE
SRDHL	SRDHL1	0.780	0.902	0.609
	SRDHL2	0.869		
	SRDHL3	0.884		
	SRDHL4	0.815		
	SRDHL5	0.680		
	SRDHL6	0.621		
TDOHI	TDOHI1	0.801	0.824	0.609
	TDOHI2	0.758		
	TDOHI3	0.782		

SRDHL, Self-rated Digital Health Literacy; TDOHI, Trust Degree of Online Health Information; CR, composite reliability; AVE, average variance extracted.

#### 3.4.3 Criterion validity

To verify the criterion validity of the DHLA, its correlation with the eHEALS was examined because eHEALS is also a commonly used method to assess DHL at present. The findings revealed a statistically significant positive correlation between the two scales (*r* = 0.720, *p* < 0.01; Table 5).

**Table 5:** Correlation between the DHLA and eHEALS.

	DHLA	eHEALS
DHLA	1	
eHEALS	0.720*	1

* means *p* < 0.01, DHLA, Digital Health Literacy Assessment; eHEALS, eHealth Literacy Scale.

### 3.5 Group comparisons

One-way ANOVA between male and female students on the total score and the scores of both two dimensions of the DHLA were also conducted. The scores for female students were lower than those for male students on both the total score (37.2 ± 6.7 vs. 38.5 ± 7.5) and the scores of each of the two dimensions (23.6 ± 4.1 vs. 24.0 ± 4.7 for SRDHL, 13.6 ± 3.3 vs. 14.5 ± 3.4 for TDOHI). The dimension with the largest difference in scores was the total score. Moreover, there were no significant differences between male and female students in both the total and individual scores for the two dimensions (Table 6).

**Table 6:** Comparison between groups by sex.

Score	Male (*n* = 79) Mean (SD)	Female (*n* = 203) Mean (SD)	T	*P*
Total score	38.5 ± 7.5	37.2 ± 6.7	2.0	0.157
SRDHL	24.0 ± 4.7	23.6 ± 4.1	0.6	0.438
TDOHI	14.5 ± 3.4	13.6 ± 3.3	3.8	0.051

SRDHL, Self-rated Digital Health Literacy; TDOHI, Trust Degree of Online Health Information.

## 4 Discussion

To the utmost of the knowledge of the authors, there was no standardized assessment to measure DHL which included interactive skills (41). This study investigated the validation of the simplified Chinese version of the DHLA which consists of 10 items from two dimensions among the university students in terms of Cronbach’s alpha, split-half reliability, structural validity, convergent validity, and criterion validity. The results showed that it was applicable for evaluating the DHL of the university students in mainland China, with good internal consistency and acceptable model fit. This study may provide a useful tool for assessing DHL and further conducting interventions for the target individuals or groups in the future.

The DHLA had a good internal consistency reliability, which was consistent with the original study (21). The value of Cronbach’s alpha in this study was 0.912, which was higher than the original study’s 0.87. In detail, the values of Cronbach’s alpha of the two dimensions (items 1–6, items 7–10) were 0.913 and 0.830, respectively, while the split-half reliability of the scale was 0.828.

The results of the KMO and Bartlett’s tests showed that the DHLA was suitable for factor analysis. However, the attribution of DHLA factors in the simplified Chinese version was inconsistent with the expected original version of the DHLA scale. Through the EFA, this study found that it was reasonable to retain two factors, which was different from the original scale with three factors. Moreover, the CFA results showed that the scale structure validity of the two dimensions and the degree of model fitting were acceptable. CFA used in this study could disprove the models or hypotheses effectively yet the results may also indicate potential adjustments that should be studied further in future analyses (42). All the fit indexes in the CFA were within the acceptable range which showed that the internal structure of the scale was relatively stable among the university students. It may be related to the difference in the meaning of “folk prescriptions” in the context of Taiwan Province and mainland China (43).

Compared to the original study, this study added convergence validity that could test the degree of correlation between items that belonged to the same variable (44). The standardized factor loadings of each item was more than 0.5. The CR of the two dimensions of the DHLA were both more than 0.70 and the AVE were both more than 0.50, indicating that the items belonging to different dimensions were highly correlated and each dimension could evaluate the content of interest. The correlation coefficient of criterion validity (r) was 0.720 (*p* < 0.01) indicating that the DHLA could measure DHL accurately.

The results of this study also showed that the average DHLA score of male students was higher than that of female students, but there was no statistically significant correlation between DHLA score and sex. Previous studies have shown inconclusive evidence regarding whether sex was statistically significantly correlated with DHL (45–47).

The study had some limitations. Firstly, due to the COVID-19 pandemic, the survey was conducted online using snowball sampling, which may increase the possibility of sampling bias and selection bias. Students who had no access to the internet or missed the survey period could not participate in the data collection. Secondly, this study collected information among the university students who generally had higher education levels and had more exposure to the internet, which may have an impact on the results of the DHLA. Future studies should include more age groups and education levels to eliminate this limitation. Thirdly, due to the geographical constraints and cultural differences, whether the DHLA scale could be extended to other countries and regions remains to be verified.

## 5 Conclusion

This study is the first to use the simplified Chinese DHLA to evaluate DHL among Chinese university students, while the high reliability and validity of the scale indicated it was acceptable as a new measurement tool to assess an individual’s DHL. Future research should attempt to examine the acceptability of this instrument in other regions and among different populations to obtain wider applications.

## Data availability statement

Under reasonable requirements, the data and material of this study can be obtained from the corresponding author.

## Ethics statement

The studies involving humans were approved by Shandong University Institutional Ethics Committee. The studies were conducted in accordance with the local legislation and institutional requirements. Written informed consent for participation in this study was provided by the participants’ legal guardians/next of kin. Written informed consent was obtained from the individual(s) for the publication of any potentially identifiable images or data included in this article.

## Author contributions

LN: Conceptualization, Data curation, Formal analysis, Funding acquisition, Investigation, Methodology, Resources, Software, Validation, Writing – original draft. JZ: Conceptualization, Methodology, Writing – review & editing. LP: Formal analysis, Investigation, Writing – review & editing. MP: Software, Supervision, Writing – review & editing. JW: Methodology, Project administration, Resources, Software, Writing – review & editing. YZ: Software, Supervision, Writing – review & editing. RC: Resources, Validation, Visualization, Writing – review & editing. HL: Conceptualization, Data curation, Formal analysis, Writing – review & editing. XX: Conceptualization, Visualization, Writing – review & editing. BS: Conceptualization, Writing – review & editing. FK: Conceptualization, Data curation, Funding acquisition, Investigation, Methodology, Project administration, Resources, Supervision, Writing – review & editing.

## Glossary

DHLA: Digital Health Literacy Assessment
DHL: Digital Health Literacy
CR: Composite reliability
AVE: Average variance extracted
SRDHL: Self-rated Digital Health Literacy
TDOHI: Trust Degree of Online Health Information
RMSEA: Root mean square error of approximation
GFI: Goodness-of-fit index
AGFI: Adjusted goodness of fit index
CFI: Comparative fit index
eHL: Electronic health literacy
DHLI: Digital Health Literacy Instrument
DHTL-AQ: Digital Health Technology Literacy Assessment Questionnaire
eHEALS: eHealth Literacy Scale
KS: Kolmogorov–Smirnov
KMO: Kaiser-Meyer-Olkin
EFA: Exploratory factor analysis
CFA: Confirmatory factor analysis
SD: Standard deviation
