# Addendum: Validation of the digital health literacy assessment among the university students in China

**DOI:** 10.3389/fpubh.2024.1388818

**Published:** 2024-04-05

**Authors:** Limei Nie, Jiajia Zhao, Lutong Pan, Mingli Pang, Jieru Wang, Yue Zhou, Rui Chen, Hui Liu, Xixing Xu, Baochen Su, Fanlei Kong

**Affiliations:** ^1^Centre for Health Management and Policy Research, School of Public Health, Cheeloo College of Medicine, Shandong University, Jinan, China; ^2^NHC Key Lab of Health Economics and Policy Research, Shandong University, Jinan, China; ^3^Institute of Health and Elderly Care, Shandong University, Jinan, China; ^4^Department of Mathematics, College of Art and Science, New York University, New York City, NY, United States

**Keywords:** digital health literacy assessment, reliability, validity, university students, China

In the published article, there was missing information. Information about the whole items of scale used in the study is now provided as Supplementary Table 1: the English and simplified Chinese version of Digital Health Literacy Assessment (DHLA) scale.

**Supplementary Table 1:** The English and simplified Chinese version of Digital Health Literacy Assessment (DHLA) scale 数字健康素养评估量表.

	**Scale items (量表条目)**	**Option (答案选项)**
Self-rated Digital Health Literacy (自评数字健康素养)	1. My ability to operate a computer/smart phone to search the online information I need (我操作电脑/智能型手机上网搜寻我所需资料的能力).	①Not good at all (非常不好) ②Not good (不好) ③Average (一般) ④Good (一般) ⑤Very good (非常好)
	2. My ability to search for the online health/disease related information (我上网搜寻健康/疾病相关信息的能力).	①Not good at all (非常不好) ②Not good (不好) ③Average (一般) ④Good (好) ⑤Very good (非常好)
	3. My ability to find the online health/disease related information (我上网找资料了解健康问题/疾病的能力).	①Not good at all (非常不好) ②Not good (不好) ③Average (一般) ④Good (好) ⑤Very good (非常好)
	4. My ability to find the online information to answer questions about healthcare/disease treatment (我上网找信息解答健康照护/疾病治疗问题的能力).	①Not good at all (非常不好) ②Not good (不好) ③Average (一般) ④Good (好) ⑤Very good (非常好)
	5. My ability to communicate with healthcare professionals using information found online (我利用上网找到的信息跟医护人员沟通的能力).	①Not good at all (非常不好) ②Not good (不好) ③Average (一般) ④Good (好) ⑤Very good (非常好)
	6. My ability to judge the correctness or incorrectness of healthcare information found online (我判断上网找到的健康信息正确与否的能力).	①Not good at all (非常不好) ②Not good (不好) ③Average (一般) ④Good (好) ⑤Very good (非常好)
Trust Degree of Online Health Information ②(网络健康信息信任度)	7. My trust degree of the health-related information found online (我对于上网找到的健康相关信息的信任度).	①Strongly distrust (非常不信任) ②Distrust (不信任) ③Generally (一般) ④Trust (信任) ⑤Strongly trust (非常信任)
	8. My trust degree of the online health-related information provided by doctors (我对于上网找到的、由医生提供的健康相关信息的信任度).	①Strongly distrust (非常不信任) ②Distrust (不信任) ③Generally (一般) ④Trust (信任) ⑤Strongly trust (非常信任)
	9. My trust degree of online health-related information provided by hospitals (我对于上网找到的、由医院提供的健康相关信息的信任度).	①Strongly distrust (非常不信任) ②Distrust(不信任) ③Generally(一般) ④Trust(信任) ⑤Strongly trust(非常信任)
	10. My trust degree of online health-related information related to folk prescriptions (我对于上网找到与民间偏方有关的健康相关信息的信任度).	①Strongly distrust(非常不信任) ②Distrust (不信任) ③Generally (一般) ④Trust (信任) ⑤Strongly trust (非常信任)

The DHLA scale consists of 10 questions using a 5-point Likert scale. Answer options from 1 (Not good at all) to 5 (Very good) for 1–6 items and 1 (Strongly distrust) to 5 (Strongly trust) for 7–10 items. The total score ranges from 10 to 50. Higher scores indicate higher levels of digital health literacy.

